# T105. FACTOR ANALYSES OF SUCCESSIVE ASSESSMENTS BY MULTIPLE SCALES HAVE A CONSISTENT STRUCTURE IN A COHORT OF FIRST EPISODE PSYCHOSES

**DOI:** 10.1093/schbul/sby016.381

**Published:** 2018-04-01

**Authors:** Alexandra Berry, Max Marshall, Max Birchwood, Shon Lewis, Samei Huda, Alison Yung, Richard Drake

**Affiliations:** 1 University of Manchester; 2 University of Warwick

## Abstract

**Background:**

Depending on the nature of their items factor analyses of different scales impose different structures on the underlying psychopathological dimensions, so a broader range of scale items should be more revealing. Few studies repeat analyses over successive interviews to investigate whether psychopathology has a consistent structure or evolves, especially after first presentations when the illness is most plastic and cohorts are unselected by chronicity.

**Methods:**

A cohort was recruited from consecutive presentations aged 16–35 to NHS Early Intervention in Psychosis services from 14 catchments over 5 years during the National EDEN project. All met DSM IV-R criteria for schizophrenia spectrum psychoses, brief or substance induced psychoses, mania or severe depression with psychosis. At recruitment, after 6 and 12 months each was assessed with Positive and Negative Symptom Scale (PANSS), Calgary Depression Scale (CDS), Young’s Mania Rating Scale (MRS) and Birchwood’s Insight Scale (IS). At each point principal axis factoring with oblique (Promax) rotation included all scale items simultaneously, apart from using total scores for IS. Items below communality thresholds were excluded and the analyses repeated until stable solutions were achieved with fit metrics meeting conventional thresholds. Factor solutions were selected using breaks in the scree plot and eigenvalues>1.0.

**Results:**

1003 met diagnostic criteria and 948 provided data. Each time point produced 6 factors featuring consistent items: psychosis (PANSS delusions, hallucinations, suspicion, stereotyped thinking & bizarre ideation; MRS grandiose content); excitement/mania/disorganisation (PANSS agitation; MRS elation, overactivity, pressured and disorganised speech); hostility/suspiciousness (PANSS hostility, uncooperativeness & impulsive irritability; MRS irritability & aggression); depression/anxiety (PANSS anxiety, guilt, depression; CDS subjective & objective depression, guilt & guilty ideas of reference, hopelessness, self-depreciation, suicidality, early waking); negative symptoms (PANSS blunting, emotional & social withdrawal, poor rapport, poverty of speech, retardation and avolition), and poor insight (PANSS insight, MRS insight, IS total). Depression explained 29–32% of variance at different stages, Psychosis 28–29%, Negative 25–26%, Excitement 19–24%, Hostility 16–23% and Poor Insight 16–23%.

**Discussion:**

The cohort, recruited from consecutive presentations, included a full range of psychoses in sufficient numbers to factor analyse the scales’ 51 parameters. There was evidence for 6 factors slightly different from the traditional 3 SAPS/SANS (Scales for the Assessment of Positive and Negative Symptoms) or 5 PANSS factors derived using chronically unwell samples with non-affective psychosis. There was more consistency than in previous first episode follow-up studies and affective and insight dimensions were more clearly defined.

